# Sacituzumab govitecan combined with anlotinib in metastatic triple negative breast cancer patient with repeated pleural effusion: a case report

**DOI:** 10.3389/fphar.2026.1800856

**Published:** 2026-09-22

**Authors:** Congcong Wang, Lulu Xu, Hao Han, Ping Sun, Huajun Qu, Jiannan Liu

**Affiliations:** Departments of Oncology, The Affiliated Yantai Yuhuangding Hospital of Qingdao University, Yantai, Shandong, China

**Keywords:** anlotinib, metastatic, pleuraleffusion, sacituzumab govitecan, triple-negative breast cancer

## Abstract

Metastatic triple-negative breast cancer (mTNBC) is an aggressive malignancy with limited targeted therapeutic alternatives and dismal long-term prognosis. Sacituzumab govitecan (SG) is a Trop-2-directed antibody-drug conjugate (ADC) conjugated to the topoisomerase I inhibitor SN-38. SG has demonstrated robust anti-tumor activity across multiple clinical trials for mTNBC. We present a hypothesis-generating case report of a 50-year-old female patient with stage III triple-negative breast cancer (TNBC) who received curative-intent surgery followed by adjuvant multimodal therapy. Fourteen months after completing adjuvant treatment, the patient developed bone-only recurrence and received local bone radiotherapy plus first-line albumin-bound paclitaxel plus carboplatin chemotherapy, followed by capecitabine maintenance until 25 April 2023. Upon successive systemic disease progression, the patient sequentially received second-line toripalimab plus gemcitabine and third-line toripalimab plus eribulin through November 2023. Cervical lymph node biopsy confirmed metastatic TNBC with negative BRCA germline status and PD-L1 tumor proportion score (TPS) < 1%. Per international and domestic clinical guidelines, single-agent SG (10 mg/kg) was initiated as fourth-line therapy. During SG monotherapy, recurrent symptomatic malignant pleural effusion developed, and oral anlotinib 10 mg (days 1–14 every 21-day cycle) was added to the treatment regimen starting 18 July 2024. As of 4 February 2025, the patient completed 7 cycles of SG monotherapy and 9 cycles of sequential SG plus anlotinib combination treatment. Clinically, no recurrent pleural effusion was observed after anlotinib initiation; measurable pulmonary, hepatic, and cervical nodal metastases exhibited sustained shrinkage alongside declining serum tumor markers, yielding a 12-month progression-free survival (PFS). Grade 1–2 treatment-emergent adverse events included leukopenia, neutropenia, diarrhea, and fatigue, all manageable with supportive care. To our knowledge, this is the first descriptive case documenting sustained disease control following SG plus anlotinib combination in heavily pretreated mTNBC complicated by recurrent malignant pleural effusion. It should also be noted that although the timing of clinical symptom improvement coincided with the initiation of anlotinib, this single case cannot differentiate whether the clinical benefit observed derived from the potential synergistic effect of the two agents or the monotherapeutic effect of anlotinib alone. Without validation from phase II/III prospective clinical trials, this case only generates a preliminary hypothesis that SG combined with anlotinib may represent a potential salvage therapeutic strategy for heavily pretreated advanced TNBC patients complicated by refractory malignant pleural effusion or localized progression under single-agent SG. Further prospective clinical research is required to verify the synergistic activity of this regimen.

## Introduction

Triple negative breast cancer (TNBC) is a special type of breast cancer, characterized by the lack of expression of estrogen receptor (ER), progesterone receptor (PR) and human epidermal growth factor receptor 2 (HER2). Due to the absence of these receptors, TNBC cannot be treated by traditional hormone therapy or HER2 targeted therapy, and treatment options are relatively limited, mainly relying on chemotherapy. New therapeutic drugs, such as poly ADP ribose polymerase (PARP) inhibitors, immune checkpoint inhibitors (ICIs) and antibody-drug conjugates (ADC), have been approved for the treatment of TNBC, improving patient prognosis (Robson et al., 2017; Cortes et al., 2020; Modi et al., 2022).

As the first approved Trop-2 ADC drug in the world, sacituzumab govitecan (SG) has significantly changed the treatment pattern of patients with metastatic TNBC. Sacituzumab govitecan (SG) is a Trop-2-targeted antibody-drug conjugate carrying SN-38, a potent topoisomerase I inhibitor, which has been approved for heavily pretreated metastatic triple-negative breast cancer (Kumari et al., 2025).

In the phase III ASCENT trial, compared to traditional chemotherapy, SG significantly prolonged progression free survival (PFS) and overall survival (OS) in patients receiving second-line and above treatment (Bardia et al., 2021). Among patients receiving SG, the median PFS increased from 1.7 months to 5.6 months, and the median OS increased from 6.7 months to 12.1 months. These results highlight the significant efficacy of SG in the treatment of advanced TNBC. Moreover, the efficacy of SG is not affected by prior treatment, making it the preferred treatment option for metastatic TNBC patients who had received two or more lines of chemotherapy.

Malignant pleural effusion can occur in all malignant tumors, seriously reducing the quality of life of patients. Pleural metastasis of breast cancer is the second major cause of malignant pleural effusion, second only to lung cancer, which often indicates poor prognosis. Anlotinib is a multi-target tyrosine kinase inhibitor that reduces tumor angiogenesis by inhibiting vascular endothelial growth factor receptors, thereby lowering vascular permeability and effectively reducing the formation of malignant pleural effusion. In a randomized, double-blind, multicenter phase II clinical trial, anlotinib significantly prolonged progression-free survival (PFS) and overall survival (OS) compared with placebo in patients with small cell lung cancer (SCLC) complicated with malignant pleural effusion who had received at least two lines of prior chemotherapy (Liu et al., 2021).

In this report, we describe a case of advanced TNBC patient with repeated pleural effusion was treated with SG and arotinib after multi line treatment, and achieved good therapeutic effect. We explicitly frame this case as hypothesis-generating rather than definitive evidence of combination synergy.

## Case report

In October 2018, a 50-year-old woman with no relevant past medical history was diagnosed with stage III TNBC and was treated with surgery, adjuvant chemotherapy, and radiotherapy. Postoperative pathology showed TNBC, pT2N3M0 stage IIIc. The selected adjuvant chemotherapy regimen is 4 cycles of epirubicin and cyclophosphamide, followed by 4 cycles of docetaxel. The patient has started regular follow-up after radiotherapy.

On 25 August 2020, the patient suffered from back pain for more than a month. Upon further examination, bone metastasis was found and disease recurrence was considered. In September 2020, local bone radiotherapy and first-line treatment were performed, with a regimen of albumin paclitaxel combined with carboplatin, for a total of 6 cycles. Subsequent maintenance treatment with capecitabine will continue until 25 April 2023. And zoledronic acid was given to prevent bone adverse events. During the follow up examination, the condition remained stable. Subsequently, due to repeated disease progression, the patient was treated with second-line Toripalimab combined with Gemcitabine and third line Toripalimab combined with Elibulin until November 2023.

In December 2023, the patient presented with recurrent cough and productive sputum. PET/CT performed on 20 December 2023 revealed widespread metastatic lesions: multiple lymph node metastases (cervical, mediastinal, bilateral hilar, and retroperitoneal), bilateral pulmonary metastases with carcinomatous lymphangitis, multiple hepatic metastases, and extensive skeletal metastases (Figure 1).

**FIGURE 1:**
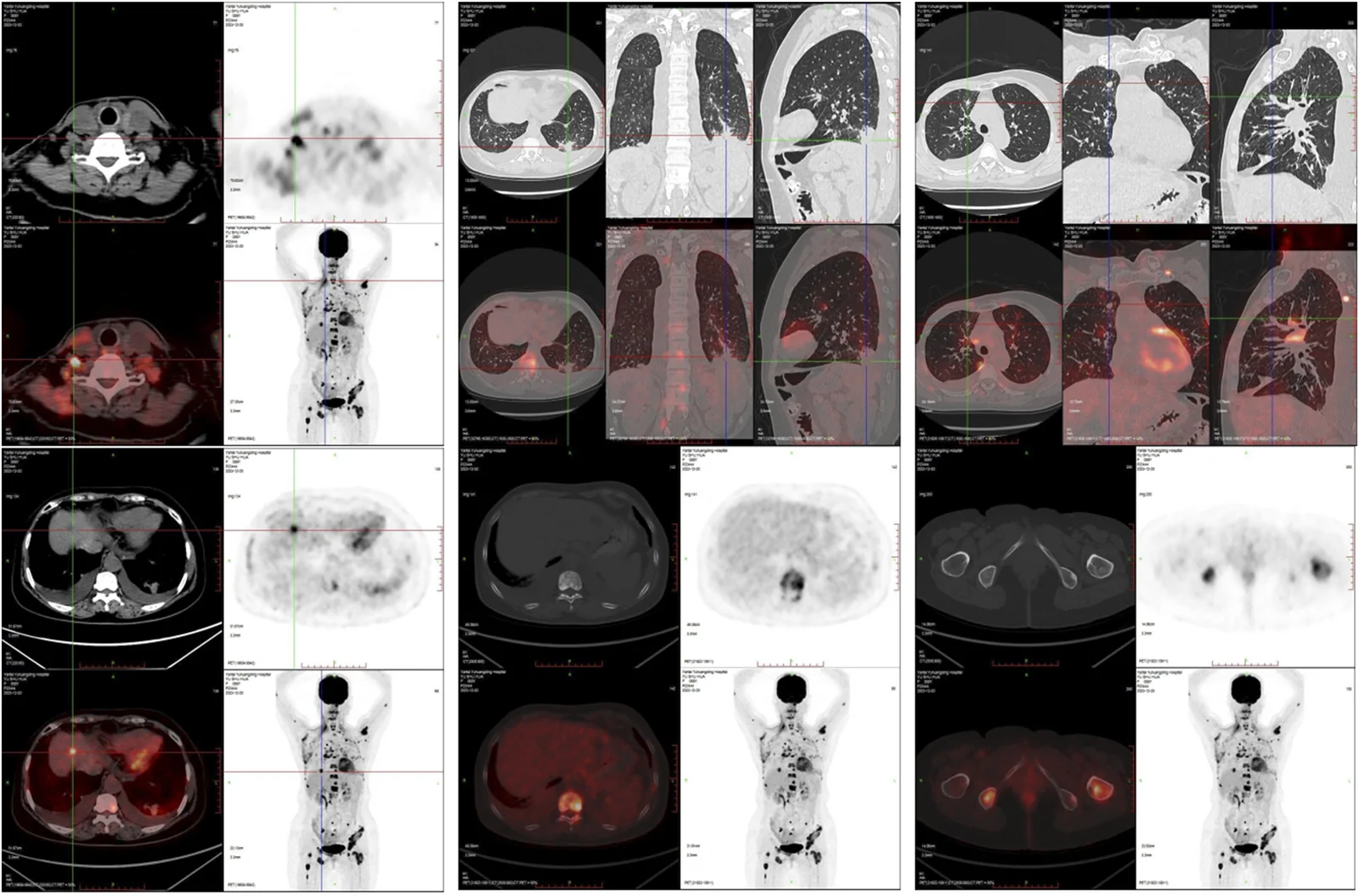
Baseline PET/CT imaging acquired 20 December 2023 prior to fourth-line SG initiation.

Ultrasound-guided biopsy of the left cervical lymph node was performed on 6 January 2024. Histopathological examination demonstrated carcinoma infiltration within fibrous tissue, consistent with breast cancer metastasis based on clinical history and immunohistochemical (IHC) profile. IHC results showed: CK7(+), GATA3 (+), TRPS1(+), ER (−), PR (−), HER2 (1+), CK5/6 (−), Ki-67 (approximately 40%+). PD-L1 status (SP263 assay) revealed tumor proportion score (TPS) < 1% and combined positive score (CPS) < 1. Blood BRCA gene testing returned negative.

Fourth-line therapy was initiated on 17 January 2024. Sacituzumab govitecan (SG) 540 mg administered on days 1 and 8 of a 21-day cycle, combined with denosumab 120 mg subcutaneously every 2–3 months.

Follow-up chest CT on 8 March 2024 demonstrated significant progression of bilateral pleural effusions. Following exclusion of alternative etiologies such as infection, cardiogenic effusion and hypoalbuminemia, bilateral chest tube drainage was conducted.

1. Infectious etiology: Repeated blood routine, procalcitonin and pleural fluid culture showed no signs of bacterial or fungal infection. No fever, cough with purulent sputum or other infectious manifestations were observed throughout treatment, and anti-infective therapy was never administered.
2. Cardiogenic effusion: Serial cardiac ultrasound, serum BNP and physical examination ruled out heart failure as a contributing factor.
3. Hypoalbuminemia: The patient’s serum albumin level was consistently 32.58–39.92 g/L during the relevant period, and there was no evidence of nephrotic syndrome or malnutrition.; no albumin infusion was needed to relieve effusion.

Pleural fluid cytology verified the presence of malignant cells, indicating malignant pleural effusion. Pathological analysis of pleural effusion cell block specimens reaffirmed breast cancer metastasis, with additional IHC findings: ER (−), PR (1% weakly positive), HER2(+). PD-L1 status (SP263) remained negative.

According to RECIST version 1.1 criteria, isolated progression of malignant pleural effusion without enlargement of solid target lesions and absence of new metastatic lesions belongs to progression of non-target lesions.

At the re-evaluation on 8 March 2024, all measurable solid metastatic lesions remained stable, serum tumor markers declined moderately (Figure 2), and no new distant metastatic lesions emerged. Therefore, we maintained SG monotherapy and adopted temporary local intrapleural cisplatin perfusion (40 mg per side) to control symptomatic effusion at that time.

**FIGURE 2:**
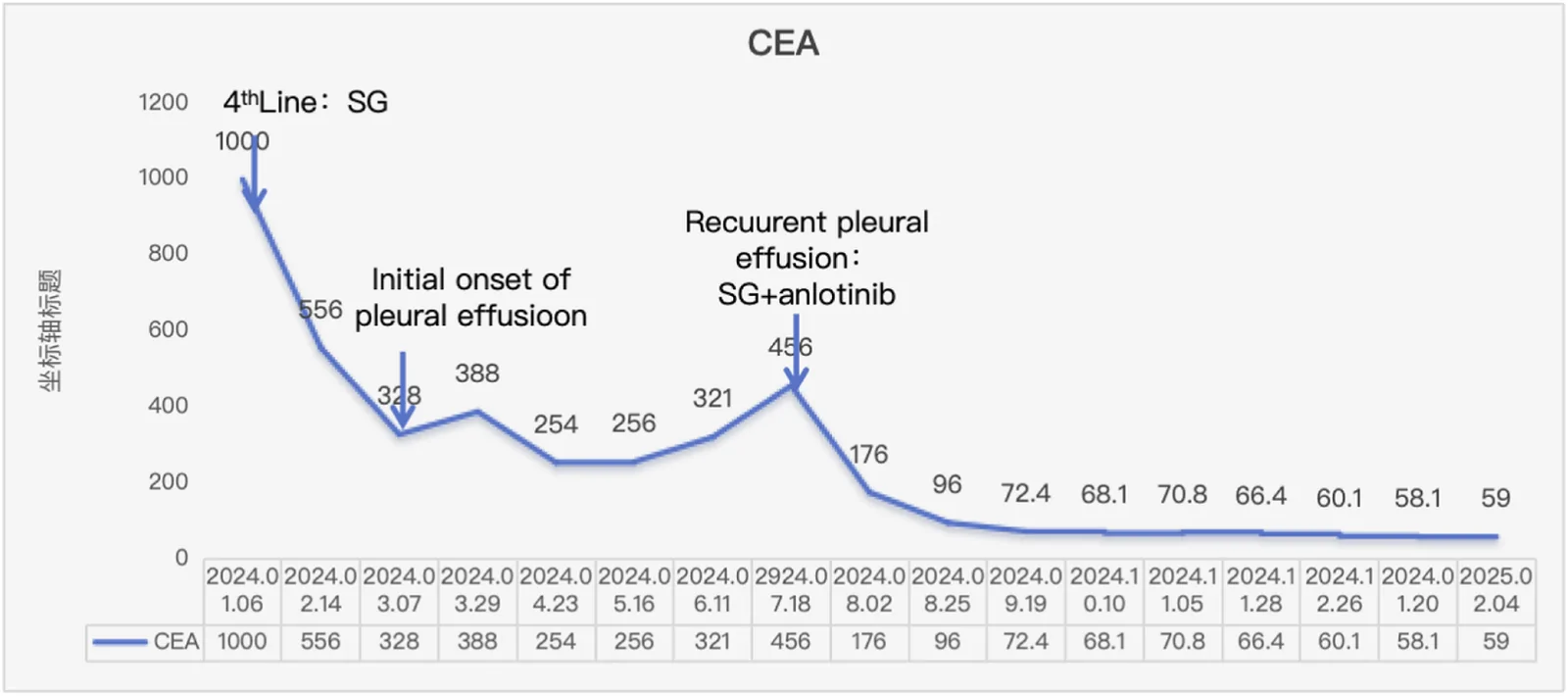
Changes in serum CEA levels during 4^th^ line therapy.

As of 18 July 2024, after 7 cycles of SG monotherapy, target lesions remained stable according to RECIST 1.1 criteria, accompanied by progressive elevation of serum carcinoembryonic antigen (CEA). Nevertheless, recurrent symptomatic malignant pleural effusion continued to progress, severely impairing the patient’s quality of life. Given that anti-angiogenic agents can improve the control of malignant serous effusions in advanced solid tumors, oral anlotinib (10 mg days 1–14, 21-day cycle) was added. After anlotinib was combined with SG starting July 2024, no further local thoracic operation was performed, and pleural effusion disappeared completely without additional local management.

By 4 February 2025, the patient had completed 7 cycles of SG monotherapy and 9 cycles of SG-anlotinib combination therapy (Figure 3). Subsequent evaluations demonstrated complete resolution of pleural effusion, sustained reduction in pulmonary/hepatic metastases and cervical lymph nodes (Partial Response) (Figure 4; Table 1), declining tumor markers (Figure 2), and progression-free survival (PFS) of 12 months.

**FIGURE 3:**
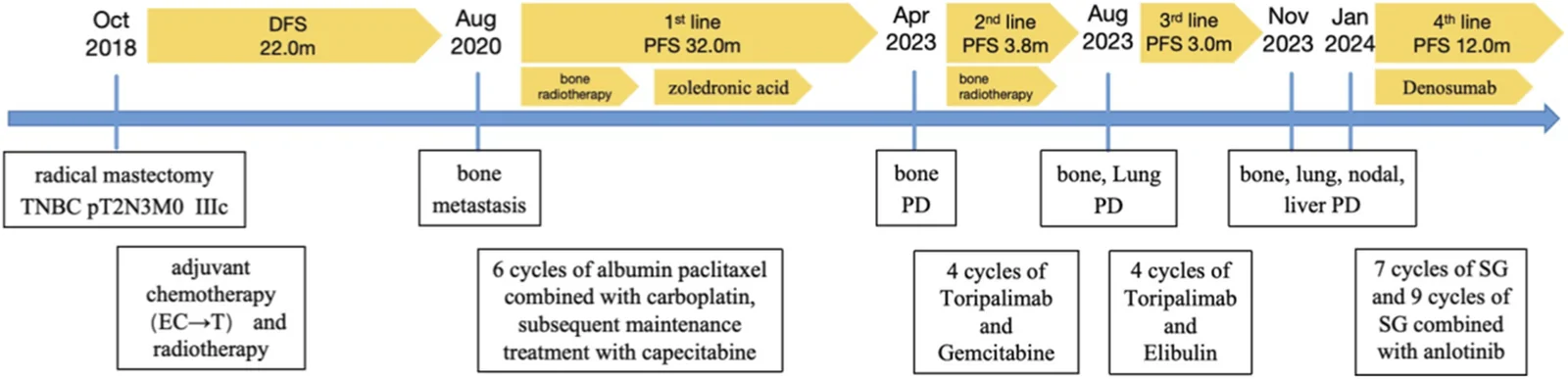
Patient’s timeline. TNBC, triple-negative breast cancer; DFS, disease-free survival; PFS, progression-free survival; PD, progressive disease.

**FIGURE 4:**
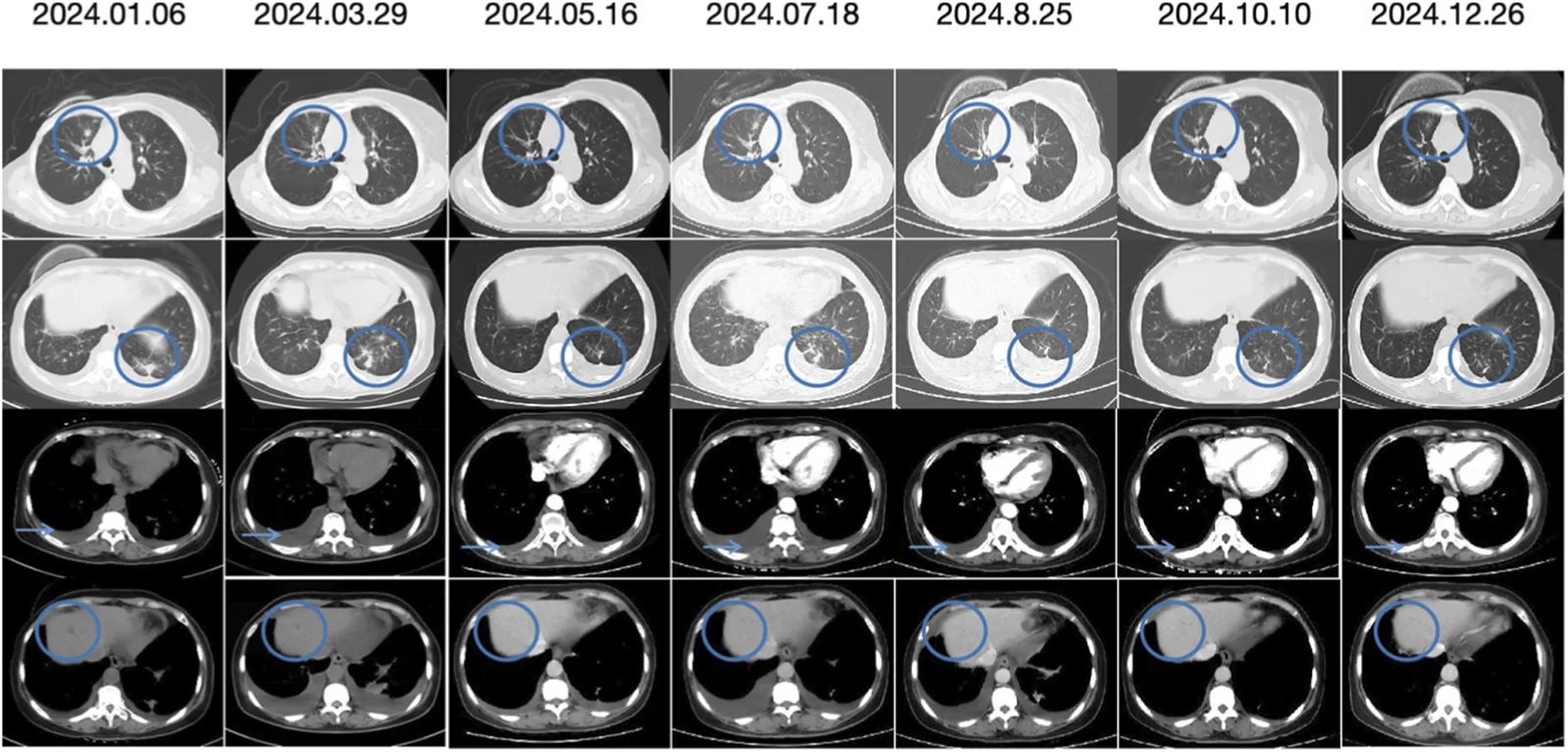
Radiological evaluation of the patient’s pulmonary and metastases.

**TABLE 1:** Changes in metastatic lesions during 4th line therapy.

​	TIME	Pulmonary metastases (center lower lobe)	Hepatic metastases	Cervical lymph nodes
SG→	2024-01	1.6cm	1.6*1.2cm	1.5*0.5cm
	2024-03	1.4cm	1.2*0.6cm	1.1*0.7cm
	2024-05	1.2cm	0.6cm	1.1*0.7cm
SG+anlotinib→	2024-07	1.4cm	0.6cm	1.2*0.4cm
	2024-08	1.0cm	0.6cm	disappeared
	2024-10	1.0cm	0.6cm	disappeared
	2024-12	1.0cm	0.6cm	disappeared

Treatment-emergent adverse events included manageable grade 2 toxicities (CTCAE v5.0): leukopenia, neutropenia, diarrhea, and fatigue. No dose reduction, treatment delay or permanent interruption of anlotinib occurred during the entire combination treatment period.

The patient remains under active follow-up with ongoing therapy.

Table 1. Changes in metastatic lesions during 4^th^ line therapy.

## Discussion

TNBC remains the molecular subtype with the worst prognosis, with a 5-year overall survival (OS) rate of only 12.2%. The patient initially presented with locally advanced TNBC but did not undergo neoadjuvant therapy due to personal preference. The recurrence and metastasis within 2 years post-surgery indicate a poor prognosis.

Following disease progression, the patient received multiple lines of chemotherapy, including regimens combined with immune checkpoint inhibitors. The first-line chemotherapy achieved a progression-free survival (PFS) of 32 months. However, subsequent second- and third-line therapies incorporating immunotherapy demonstrated limited efficacy, with a PFS of only 3 months. This suboptimal response may be associated with the patient’s PD-L1 expression of less than 1%, a known negative predictor for immunotherapy efficacy in TNBC.

Based on the findings from the ASCENT trial, sacituzumab govitecan (SG) has been established as a standard-of-care option for advanced triple-negative breast cancer (TNBC) in the second-line and beyond settings, as recommended by both domestic and international guidelines. The Chinese bridging study EVER-132–001 (Xu et al., 2023) further validated the efficacy and safety of SG in Chinese patients with advanced TNBC. Beyond the multinational, multicenter, randomized controlled phase III data, additional evidence from SG-related meta-analyses and real-world studies further supports its role as an essential therapeutic option for metastatic breast cancer.

Following fourth-line treatment with sacituzumab govitecan (SG), the patient exhibited decreased tumor markers and reduced lesion size. Despite developing pleural effusion requiring localized management, continued SG administration maintained clinical benefit, indicating favorable therapeutic efficacy of this agent.

As a multi-target tyrosine kinase inhibitor (TKI), anlotinib demonstrates dual inhibition of VEGFR2 and PDGFR-β, potentially synergistically ameliorating effusion through two distinct mechanisms: 1) reducing VEGF-mediated vascular leakage by suppressing tumor-derived VEGF secretion; 2) enhancing lymphatic drainage via PDGFR-β inhibition-mediated attenuation of interstitial fibrosis (Lin et al., 2018). This bidirectional modulation of the “vascular-stromal axis” provides novel theoretical foundations for managing malignant effusions.

The SN-38 payload of SG induces immunogenic cell death (ICD) to boost tumor antigen presentation and anti-tumor adaptive immunity (Bardia et al., 2017). Meanwhile, anlotinib repolarizes immunosuppressive tumor-associated macrophages (TAMs) via CSF-1R inhibition to remodel an immune-permissive tumor microenvironment (Yang et al., 2020). Additionally, anti-angiogenic TKIs such as anlotinib may normalize disorganized tumor vasculature and improve ADC tissue penetration into metastatic lesions (Van Der Veldt et al., 2012). We emphasize that these mechanistic synergies are purely hypothetical in this single clinical case and cannot be confirmed without controlled comparative data.

In the present case, the patient achieved remarkable efficacy with resolved recurrent pleural effusion following anlotinib combination therapy. Regarding safety profile, the primary adverse events included grade 2 leukopenia, grade 2 neutropenia, grade 2 diarrhea, and grade 1 fatigue, all of which were well-managed with supportive care.

This case study has several limitations as follows: 1) There were confounding therapeutic interventions. During fourth-line monotherapy with sacituzumab govitecan (SG), the patient developed malignant pleural effusion for the first time and received bilateral thoracic catheter drainage as well as intrapleural cisplatin perfusion. Accordingly, it is impossible to separate the independent therapeutic effect of a single intervention on intrathoracic lesions and systemic metastatic lesions. 2) Absence of controlled design: This is a single descriptive case report without a control cohort, so the exclusive efficacy of anlotinib in managing refractory malignant pleural effusion cannot be isolated. 3) Lack of prospective clinical evidence: At present, no Phase II or Phase III clinical trials have confirmed the efficacy of sacituzumab govitecan combined with anlotinib for advanced triple-negative breast cancer complicated with malignant pleural effusion.

To our knowledge, this is the first published case documenting sustained disease control with sequential SG followed by SG-anlotinib combination in heavily pretreated mTNBC complicated by refractory malignant pleural effusion. This work does not provide confirmatory evidence of combination therapeutic superiority, but generates a testable clinical hypothesis for future prospective investigation. Prospective controlled clinical trials are urgently required to quantify the synergistic anti-tumor and anti-effusion activity of SG plus anlotinib and define the optimal population for this salvage regimen.

## Data Availability

The original contributions presented in the study are included in the article/supplementary material, further inquiries can be directed to the corresponding authors.
